# Ethics and Best Practice Guidelines for Training Experiences in Global Health

**DOI:** 10.4269/ajtmh.2010.10-0527

**Published:** 2010-12-06

**Authors:** John A. Crump, Jeremy Sugarman, the Working Group on Ethics Guidelines for Global Health Training (WEIGHT)

**Affiliations:** Division of Infectious Diseases and International Health, Duke University Medical Center, Durham, North Carolina; Duke Global Health Institute, Duke University, Durham, North Carolina; Kilimanjaro Christian Medical Centre, Moshi, Tanzania; Kilimanjaro Christian Medical College, Tumaini University, Moshi, Tanzania; Berman Institute of Bioethics and Department of Medicine, Johns Hopkins University, Baltimore, Maryland; Office of Global Health, Stanford University, Stanford, California; Researcher, Bioethics and Global Health, Pune, India; Fogarty International Center, National Institutes of Health, Bethesda, Maryland; Departments of Medicine and Public Health, Stony Brook University School of Medicine, Stony Brook, New York; Emory Global Health Institute, Emory University, Atlanta, Georgia; Naval Medical Research Center Detachment, Lima, Peru; Doris Duke Charitable Foundation, New York, New York; BMJ, London, United Kingdom; Clinical Research Unit, London School of Hygiene and Tropical Medicine, London, United Kingdom; Chula Medical Research Center (ChulaMRC), Faculty of Medicine, Chulalongkorn University, Bangkok, Thailand; HIVNAT, Thai Red Cross AIDS Research Center, Bangkok, Thailand; College of Health Sciences, Makerere University, Kampala, Uganda

## Abstract

Academic global health programs are growing rapidly in scale and number. Students of many disciplines increasingly desire global health content in their curricula. Global health curricula often include field experiences that involve crossing international and socio-cultural borders. Although global health training experiences offer potential benefits to trainees and to sending institutions, these experiences are sometimes problematic and raise ethical challenges. The Working Group on Ethics Guidelines for Global Health Training (WEIGHT) developed a set of guidelines for institutions, trainees, and sponsors of field-based global health training on ethics and best practices in this setting. Because only limited data have been collected within the context of existing global health training, the guidelines were informed by the published literature and the experience of WEIGHT members. The Working Group on Ethics Guidelines for Global Health Training encourages efforts to develop and implement a means of assessing the potential benefits and harms of global health training programs.

## Preface

Educational institutions, foundations, and governmental and non-governmental organizations have shown a growing interest in applying their technical expertise, energy, talent, research capability, and resources to addressing global health challenges and disparities.^1^–^4^ Students increasingly request global health content in curricula and often wish to experience global health challenges firsthand.^5^–^7^ Accordingly, global health educational programs frequently include field experiences that often involve crossing international borders and during which trainees often encounter ethical challenges related to cultural and professional differences.^8^

Health science students participating in global health field experiences have been shown to be more likely to care for the poor and ethnic minorities, to change focus from sub-specialty training to primary care medicine, to report improved diagnostic skills, and to express increased interest in volunteerism, humanitarianism, and public health.^9^–^14^ For these and other trainees, such experiences may form the foundations for a career focused on or oriented toward global health or may help them to decide against such a career.^15^ By offering short-term global health field experiences, sending institutions may strengthen their position to recruit trainees interested in global health and to benefit from the appeal of such programs to funders and philanthropists.

Because global health is inherently interdisciplinary and multidisciplinary,^16^ students from a growing range of disciplines directly and indirectly related to health seek training in short-term experiences. Students also represent a range of levels and experience and may include undergraduate students, graduate students, and faculty wishing to expand their work into the global health arena. Bi-directional exchange programs offer trainees the opportunity to experience health issues in each other's environments. Experiences may vary in duration from as short as a few days to as long as 12 months and may vary considerably in quality.^17^ The goals of training experiences also vary; some can be viewed as training opportunities for the primary benefit of the trainee, whereas others claim to provide some form of service to the host or may involve research.^18^,^19^ However, little is known about the benefits and unintended consequences of global health training experiences to host institutions and host trainees and, if a component of service is anticipated, whether benefit is realized and at what cost.^20^–^22^ Global health training that benefits the trainee at the cost of the host is clearly unacceptable; mutual and reciprocal benefit, geared to achieving the program goals of all parties and aiming for equity, should be the goal.^1^ Exploitation of one partner for the benefit of another must be avoided.

Although global health training experiences offer potential benefits to trainees and to sending institutions and appear to be growing rapidly in scale, these experiences are sometimes problematic and raise ethical challenges.^1^,^18^,^23^–^25^ Such challenges include substantial burdens on the host in the resource-constrained setting; negative impact on patients, the community, and local trainees; unbalanced relationships among institutions and trainees; and concerns related to sustainability^26^,^27^ and optimal resource utilization. Although considerable attention has been given to ethical issues surrounding research conducted across international borders^28^ and under circumstances of unequal wealth or power, much less attention has been given to the ethical issues associated with education and service initiatives of global health programs and no formal ethical guidelines are available for global health training experiences. To develop ethics and best practice guidelines, we formed the Working Group on Ethics Guidelines for Global Health Training (WEIGHT). The WEIGHT members were selected by JAC and JS through a process of consultation with leaders in global health and ethics. The goal was to select members with experience and expertise with global health training and ethics from a range of perspectives and geographic locations. Of 13 initial membership invitations, 10 (77%) accepted. Those who declined were replaced by persons with similar expertise and experience to create a balanced membership.

**Summary Points**

• Academic global health programs are growing rapidly in scale and number.
• Global health curricula often include field experiences that involve crossing international and socio-cultural borders.
• Although global health training experiences offer potential benefits to trainees and to sending institutions, these experiences are sometimes problematic and raise ethical challenges.
• The Working Group on Ethics Guidelines for Global Health Training (WEIGHT) developed a set of guidelines for institutions, trainees, and sponsors of field-based global health training on ethics and best practices in this setting.
• The WEIGHT guidelines address the need for structured programs between partners; the importance of a comprehensive accounting for costs associated with programs; the goal of mutual and reciprocal benefit; the value of long-term partnerships for mitigating some adverse consequences of short-term experiences; characteristics of suitable trainees; the need to have adequate mentorship and supervision for trainees; preparation of trainees; trainee attitudes and behavior; trainee safety; and characteristics of programs that merit support by sponsors.
• To refine the guidelines, WEIGHT encourages work aimed at developing and implementing means of assessing the potential benefits and harms to institutions, personnel, trainees, patients and the community in host countries of global health training programs.

## Guideline Development Process

The international, peer-reviewed literature was searched for publications relevant to ethics of global health training and a paper was published raising ethical concerns for global health training programs.^1^ Reflecting the nascent nature of ethics research and scholarship in the area of global health training, published literature on the topic represented case reports, case series, and expert opinion. Following the formation of WEIGHT, the literature review was updated and an annotated bibliography was sent to members. The WEIGHT met in person in March 2010 in London to draft a preliminary set of ethics and good practice guidelines through group discussion around ethical issues that have arisen for individuals and institutions that send or receive trainees in global health. The guidelines were developed through a moderated workshop format. All members were given the opportunity to raise and discuss dissenting views for each recommendation. Agreement was reached by consensus. The primary goal of the guidelines is to facilitate the structuring of an ethically responsible global health training program and to discourage the implementation and perpetuation of imbalanced and inequitable global health training experiences and programs.

## Scope of the Guidelines

The guidelines are structured to address the multiple stakeholders involved with global health training experiences. The main stakeholders are host institutions, including program directors, mentors, other faculty, and support staff based at the receiving institution; trainees both foreign and local; sending institutions, including program directors, mentors, administrators, and managers; patients and the community at the host site; sending countries, including committees or councils responsible for medical and research ethics, and other health professional education; and sponsors of global health training. The guidelines are designed to apply to multiple levels of trainees, including undergraduates, graduate and medical students, post-graduate students, and others such as faculty or other professionals seeking to apply or expand their skills in the global health arena. Although the guidelines are predominantly focused on ethical issues for programs sending trainees from wealthier to less wealthy settings, many of the principals also apply to bi-directional trainee exchanges. The guidelines encompass the multiple disciplines and multiple activities that take place under the umbrella of global health including in the clinical, public health, research, and education arenas. Although these guidelines were developed in response to the global health activities of educational institutions, the principles are applicable and adaptable to informal programs and individual global health efforts. They also apply to programs of varying duration, while recognizing that duration can affect the nature of issues encountered. Although the guidelines can apply to exchange programs locally and internationally, they are not intended to address ethics issues encountered during long-term (> 1 year) global health service or by experts providing technical assistance. The WEIGHT recognizes that the evidence available to inform the guideline development process was limited and expects that the proposed approach to global health training will be refined in the future as new data are accumulated.

## Guidelines

### Sending and host institutions.

Well-structured programs seem to be the optimal means of ensuring optimal training programs in global health. Developing and maintaining well-structured programs generally involves a sustained series of communications and seems to have a common set of attributes as listed below, and may include clear delineation of roles and responsibilities of all parties, budgets, duration of attachments, participation in and distribution of written reports, and other products. We recommend that sending and host institutions should do the following:

1. Develop well-structured programs so that host and sender as well as other stakeholders derive mutual, equitable benefit including:
  a. Discuss expectations and responsibilities of both host and sending institutions and agree on terms before program implementation; the terms may be outlined within a memorandum of understanding. Revisit the expectations and responsibilities on a periodic basis;
  b. Consider local needs and priorities regarding the optimal structure of programs;
  c. Recognize the true cost to all institutions (e.g., costs of orientation, insurance, translation, supervision and mentoring, transportation, lodging, health care, administration) and ensure that they are appropriately reimbursed;
  d. Aspire to maintain long-term partnerships so that short-term experiences may be nested within them; and
  e. Promote transparency regarding the motivations for establishing and maintaining programs (e.g., to meet an educational mission, to establish a relationship that might be used to support research, to meet student need) and identifying and addressing any conflicts of interests and conflicts of obligations (e.g., to local patients, communities, or local trainees compared with the global health trainees) that may result from such a program.
2. Clarify goals, expectations, and responsibilities through explicit agreements and periodic review by
  a. Senders and hosts;
  b. Trainees and mentors; and
  c. Sponsors and recipients.
3. Develop, implement, regularly update, and improve formal training for trainees and mentors, both local and foreign regarding material that includes:
  a. Norms of professionalism (local and sending);
  b. Standards of practice (local and sending);
  c. Cultural competence, e.g., behavior (local and sending) and dealing effectively with cultural differences;
  d. Dealing appropriately with conflicts (i.e., professionalism, culture, scientific and clinical differences of approach);
  e. Language capability;
  f. Personal safety; and
  g. Implications of differential access to resources for foreign and local trainees.
4. Encourage non-threatening communication to resolve ethical conflicts as they arise in real-time and identify a mechanism to involve the host and sending institutions when issues are not readily resolved.
5. Clarify the trainees' level of training and experience for the host institution so that appropriate activities are assigned and patient care and community well-being is not compromised.
6. Select trainees who are adaptable, motivated to address global health issues, sensitive to local priorities, willing to listen and learn, whose abilities and experience matches the expectations of the position, and who will be good representatives of their home institution and country.
7. Promote safety of trainees to the extent possible (e.g., vaccinations, personal behaviors, medications, physical barriers, security awareness, road safety, sexual harassment, psychological support, insurance and knowledge of relevant local laws).
8. Monitor costs and benefits to host institutions, local trainees, patients, communities, and sponsoring institutions to assure equity.
9. Establish effective supervision and mentorship of trainees by the host and sending institution, including the selection of appropriate mentors and supervisors and facilitating communication among them.
10. Establish methods to solicit feedback from the trainees both during and on completion of the program, including exit interviews, and track the participants post-training to evaluate the impact of the experience.

### Trainees.

Trainees themselves play an important role in the quality of global health experiences. It is essential that trainees understand their responsibility in this regard, not only to ensure their personal experience is a good one, but that their actions and behaviors can have far-reaching and important implications. To help meet such responsibilities, we recommend that trainees should do the following:

1. Recognize that the primary purpose of the experience is global health learning and appropriately supervised service. The duration of the training experience should be tailored so that the burden to the host is minimized.
2. Communicate with their local mentor through official channels regarding goals and expectations for the experience before the training, and maintain communication with mentors throughout the experience.
3. Learn appropriate language skills relevant to the host's locale as well as socio-cultural, political, and historical aspects of the host community.
4. Seek to acquire knowledge and learn new skills with appropriate training and supervision, but be cognizant and respectful of their current capability and level of training.
5. Participate in the process of communicating to patients and the community about their level of training and experience so that appropriate activities are assigned and patient care and community well-being is not compromised.
6. Recognize and respect divergent diagnostic and treatment paradigms.
7. Demonstrate cultural competency (e.g., personal dress, patient privacy, culturally appropriate and inappropriate gestures, gender issues, traditional beliefs about health, truth telling, social media) and engage in appropriate discussions about different perspectives and approaches
8. Take measures to ensure personal safety and health.
9. Meet licensing standards, visa policies, research ethics review, training on privacy and security of patient information, and other host and sending country requirements.
10. Follow accepted international guidelines regarding the donation of medications, technology, and supplies.^29^,^30^
11. If research is planned as part of the training experience, develop the research plan early and in consultation with mentors, focus on research themes of interest and relevance to the host, understand and follow all research procedures of the host and sending institution, obtain ethics committee approval for the research before initiation of research, and receive appropriate training in research ethics.
12. Follow international standards for authorship of publications emanating from the global health experiences and discuss these issues and plans for presentations early in collaborations.
13. When requested, be willing to share feedback on the training experience and follow-up information on career progression.
14. When seeking global health training outside of a well-structured program, potential trainees should follow the guidelines for institutions (above) so as to maximize the benefits and minimize potential harms of such training experiences.

### Sponsors.

Sponsors of global health training programs understandably desire high quality experiences for trainees as well as minimizing any potential adverse consequences related to programs they support. By requiring recipients to be involved with high quality global health training programs as a condition of receiving funds, sponsors can play an important role in creating and maintaining such programs. Where practicable, we recommend that sponsors should do the following:

1. Promote the implementation of these guidelines.
2. Consider local needs and priorities, reciprocity, and sustainability of programs.
3. Ensure that the true costs are recognized and supported (e.g., costs of orientation, insurance, translation, supervision and mentoring, transportation, lodging, health care, administration, monitoring and evaluation).
4. Execute explicit agreements with recipients, with periodic review, to help clarify goals, expectations, and responsibilities.
5. Aim to select trainees who are adaptable, motivated to address global health issues, sensitive to local priorities, willing to listen and learn, whose abilities and experience match the expectation of the position, and who will be a good representative of their home institution and country.
6. Promote safety of trainees to the extent possible (e.g., vaccinations, personal behaviors, medications, physical barriers, security awareness, road safety, sexual harassment, psychological support, insurance, and knowledge of relevant local laws).
7. Encourage effective supervision and mentorship by the host and sending institution.
8. Require that sponsored programs comply with licensing standards, visa policies, research ethics review, training on privacy and security of patient information, and other host and sending country requirements.
9. Encourage the collection and evaluation of data on the impact of the training experiences.

## Conclusions

Global health training programs are associated with a range of ethical issues for all stakeholders. These ethics and best practice guidelines set out a range of measures designed to minimize the pitfalls of such programs. It is hoped that these guidelines will be used to reassess and improve existing programs, be applied in the design of new programs, and, where necessary, promote the discontinuation of programs or activities that cannot meet basic practices described in these guidelines.

Although these guidelines are based on a range of published data and the unpublished experience of WEIGHT members in consultation with stakeholders, they have limitations. The principal limitation is the lack of available systematic data collected within the context of existing global health training programs reflecting the scope of programs and challenges experienced by partners. WEIGHT encourages work aimed at developing and implementing means of assessing the potential benefits and harms to institutions, personnel, trainees, patients, and the community in host countries of global health training programs. Data from such assessments would inform and support future refinement of these guidelines. Although efforts were made to ensure that WEIGHT represented a range of perspectives and geographic locations, membership could be further expanded to include other groups such as trainees.
